# Overexpression of Calcium-Permeable Glutamate Receptors in Glioblastoma Derived Brain Tumor Initiating Cells

**DOI:** 10.1371/journal.pone.0047846

**Published:** 2012-10-23

**Authors:** Michael C. Oh, Joseph M. Kim, Michael Safaee, Gurvinder Kaur, Matthew Z. Sun, Rajwant Kaur, Anna Celli, Theodora M. Mauro, Andrew T. Parsa

**Affiliations:** 1 Department of Neurological Surgery, University of California San Francisco, San Francisco, California, United States of America; 2 Department of Dermatology, University of California, San Francisco, San Francisco, California, United States of America; University of Michigan School of Medicine, United States of America

## Abstract

Glioblastoma multiforme is the most malignant type of primary brain tumor with a poor prognosis. These tumors consist of a heterogeneous population of malignant cells, including well-differentiated tumor cells and less differentiated cells with stem cell properties. These cancer stem cells, known as brain tumor initiating cells, likely contribute to glioma recurrence, as they are highly invasive, mobile, resistant to radiation and chemotherapy, and have the capacity to self-renew. Glioblastoma tumor cells release excitotoxic levels of glutamate, which may be a key process in the death of peritumoral neurons, formation of necrosis, local inflammation, and glioma-related seizures. Moreover, elevated glutamate levels in the tumor may act in paracrine and autocrine manner to activate glutamate receptors on glioblastoma tumor cells, resulting in proliferation and invasion. Using a previously described culturing condition that selectively promotes the growth of brain tumor initiating cells, which express the stem cell markers nestin and SOX-2, we characterize the expression of α-amino-3-hydroxy-5-methyl-4-isozolepropionic acid (AMPA)-type glutamate receptor subunits in brain tumor initiating cells derived from glioblastomas. Here we show for the first time that glioblastoma brain tumor initiating cells express high concentrations of functional calcium-permeable AMPA receptors, compared to the differentiated tumor cultures consisting of non-stem cells. Up-regulated calcium-permeable AMPA receptor expression was confirmed by immunoblotting, immunocytochemistry, and intracellular calcium imaging in response to specific agonists. Our findings raise the possibility that glutamate secretion in the GBM tumor microenvironment may stimulate brain tumor derived cancer stem cells.

## Introduction

Glioblastoma multiforme (GBM) is the most aggressive form of primary brain tumor with a median survival of approximately 14 months, despite aggressive surgical resection followed by adjuvant therapies [1]. Poor survival is directly related to high recurrence rates that reflect the highly invasive and mobile nature of glioma cells [2]–[4]. Brain tumor initiating cells (BTICs), sometimes referred to as brain tumor or cancer stem cells, have been proposed to be responsible not only for tumor progression and initiation, but also for recurrences following surgery and adjuvant therapies [5]–[8]. BTICs are able to migrate from the primary tumor and invade into normal surrounding brain, forming microsatellites of malignant cells that can evade a surgical resection. Moreover, BTICs are more resistant to radiation [9]–[11] and chemotherapy [12], [13] compared to the non-BTICs in the tumor mass. Accordingly, a better understanding of the molecular makeup of BTICs and the key differences between BTICs and non-BTICs could lead to powerful novel therapies to treat GBM by preventing recurrences.

Several studies support the premise that glioma cells release excitotoxic levels of glutamate [14]–[17] and can express glutamate receptors [18]–[22]. The release of glutamate from glioma cells has been attributed to the lack of expression of GLT-1 glutamate transporter as well as mislocalization of another glutamate transporter GLAST to the nuclear membranes in glioma cells [23], which are the two predominant glutamate transporter subtypes expressed in glial cells of normal brain [24], [25]. Moreover, active glutamate release by glioma cells is mediated by the cysteine-glutamate exchanger (system x_c_^-^) [23], [26]. Increased glutamate concentration in the tumor milieu can act in paracrine and autocrine manner to activate glioma cells that express glutamate receptors. Consistent with this notion, many have proposed that the increased glutamate concentration in the tumor, as well as in the peritumoral tissues, is responsible for the formation of necrosis in malignant gliomas [27], excitotoxic death of surrounding normal neurons [17], [28]–[31], glioma-related seizures [32]–[34], proliferation of tumor cells [19], [28]–[31], [35]–[37], and increased invasion and migration of glioma cells [19], [38], [39].

There are a variety of glutamate receptor types. Glioma cells actively express ionotropic glutamate receptors, including α-amino-3-hydroxy-5-methyl-4-isozolepropionic acid (AMPA), kainate, and N-methyl-D-aspartate (NMDA) receptors [18]–[22]. AMPA receptors, the most common excitatory ion channels in the brain, are made up of four different subunits named GluR1 through GluR4. Different subunit combinations form a tetramer to form a functional channel. The presence of GluR2 or GluR3 subunits results in calcium-impermeable AMPA receptors, while GluR1 and GluR4 subunits form calcium-permeable channels. Interestingly, calcium-permeable AMPA receptors lacking GluR2 subunits have been shown to be expressed in GBM cells [19]. Furthermore, activation of these receptors has been shown to promote increased mobility and proliferation of glioma cells, further contributing to their increased invasive potential [19], [36]. However, studies analyzing the expression of glutamate receptors in GBM BTICs have not been performed. In this study, we selectively cultured adherent stem cell marker-enriched BTICs from freshly resected GBM tissues [40] and characterized the expression of AMPA receptors compared to differentiated GBM cultures derived from patient-matched tumor tissues. We show here for the first time that ionotropic AMPA receptors, specifically the calcium-permeable AMPA receptors containing GluR1 and GluR4 subunits, are highly expressed in GBM BTICs but not in differentiated cultures derived from the same tumor tissues. Furthermore, we confirm that these receptors are expressed on the surface membranes of BTICs and allow calcium influx when activated by specific agonists, indicating the functional capability of these receptors in GBM BTICs.

## Materials and Methods

### Selective Culturing of Adherent GBM BTICs

GBM tissues were obtained from surgeries performed at University of California San Francisco (UCSF) by the corresponding author according to the protocol approved by the Committee on Human Research. Informed written consents were obtained from patients prior to the use of their tumor tissues for research, following the guidelines set by the UCSF Brain Tumor Research Center. Patients underwent surgical resection of their brain tumor as part of standard management and not explicitly for this study.

Selective culturing of BTICs from GBM was carried out as previously described by Pollard and colleagues [40]. Intra-operative navigation was used during surgery to obtain the tumor tissues for cultures. We specifically targeted the gadolinium-enhancing tissues of the tumor and avoided necrotic or non-enhancing areas. Freshly resected tumors confirmed as GBM by pathology were dissociated in papain (Worthington-Biochemical) for 30 minutes in 37°C water bath, and the resulting cell suspension was passed through a 50 µm cell strainer. After coating the culture flasks with 10 µg/ml laminin (Sigma) for 3 hours in 37°C incubator, half of the dissociated tumor cells were plated in NeuroCult NS-A Basal medium (Stem Cell Technologies) supplemented with N-2 supplement, B27, epidermal growth factor (EGF, 20 ng/ml), fibroblast growth factor-2 (FGF-2, 20 ng/ml), L-glutamine, pyruvate, and 1% penicillin/streptomycin (see Supplemental Data [40]). After cells were grown to near confluence while media was replaced every 3–5 days, cultures were split 1∶3 to 1∶5. After three to four passages, cells were tested for the presence of stem cell markers, nestin and Sox-2, by immunoblotting as described in the following section.

The other half of the dissociated tumor cells was used to create non-BTIC differentiated GBM cultures to be used as comparison to GBM BTIC cultures. Differentiated GBM cells were cultured in in RPMI-1640 media with 25 mM HEPES, 2 g/L sodium bicarbonate, 2 g/L glucose, 0.3 g/L L-glutamine media, 10% fetal bovine serum (FBS), 1% non-essential amino acids, 1% sodium pyruvate, and 1% penicillin/streptomycin. The established GBM cell line U251 was grown in DMEM (Dulbecco's Modified Eagle Medium) with 4.5 g/L glucose, 0.548 g/L L-glutamine, 3.7 g/L NaHCO_3_+10% FBS +1% penicillin/streptomycin.

### Immunoblotting

Western blot analysis was performed for cell lysates harvested from cultured GBM cells. Cells were lysed in lysis buffer [in mM: 20 Tris-HCl (pH 7.6), 150 sodium chloride, 1 EDTA, 1 EGTA, 2.5 sodium pyrophosphate, 1 beta-glycerophosphate, 1 sodium orthovanadate, 0.1 phenylmethylsulfonyl fluoride; 1% Triton X-100, 1 µg/ml leupeptin, 1 tablet of PhosSTOP phosphatase inhibitor cocktail (Roche, Indianapolis, IN), and 1 tablet of Complete protease inhibitor cocktail (Roche, Indianapolis, IN) in 10 mL]. Lysates were cleared of insoluble material by centrifugation at 14,000 rpm for 20 min. Loading concentrations were then normalized to bovine serum albumin (BSA) standards using the BCA protein assay (Thermo Scientific, Rockford, IL). 40 µg of protein samples was heated to 90°C in 5X SDS sample buffer [10% sodium dodecyl sulfate, 1% β-mercaptoethanol, 20% glycerol, 0.2 mM Tris (pH 6.8), 0.05% bromophenol blue] for 10 minutes, electrophoresed through 4–20% Tris-Glycine gels (Invitrogen, Carlsbad, CA), and resolved proteins were transferred to Immun-Blot PVDF membrane (BioRad, Hercules, CA). Membranes were blocked with 5% milk and 3% BSA dissolved in Tris-buffered saline (TBS) containing 0.05% Tween-20. Blots were then incubated overnight at 4°C with the primary antibody in TBS containing 5% milk and 0.05% Tween-20. After washing, membranes were incubated for 1 hour at room temperature with secondary antibody conjugated to horseradish peroxidase (Santa Cruz Biotechnology, Santa Cruz, CA). Blots were developed with Amersham ECL Western Blotting Detection reagent (GE Healthcare, Buckinghamshire, UK). Primary antibodies included antibodies against GluR1 C-terminus (Millipore, polyclonal), GluR2 N-terminus (Invitrogen, monoclonal), GluR4 N-terminus (Epitomics, monoclonal), nestin (Abcam, monoclonal), Sox-2 (Abcam, monoclonal), and GAPDH (Cell Signaling, monoclonal). All of these primary antibodies have been confirmed to react with either human or mouse proteins, and thus, we used normal mouse brain homogenized in homogenization buffer (0.32 M sucrose, 2 mM EDTA, 10 mM HEPES pH 7.4, Complete protease inhibitor cocktail, and PhosSTOP phosphatase inhibitor cocktail) as a positive control.

### Immunocytochemistry

Cells were plated on 20×20 mm glass cover slips at 2.5×10^5^ cells per cover slip. When cells reached 75–80% confluence, they were fixed in 4% paraformaldehyde for 15 minutes, then washed 3 times in 10% FBS in phosphate buffered saline (PBS). Cells stained for intracellular markers (α-tubulin; Cell Signaling, monoclonal) were permeabilized in 0.5% Triton X-100 (Sigma Aldrich) in 10% FBS-PBS for 10 minutes at 4°C, then washed 3 times. Cells were blocked for 1 hour in 10% FBS-PBS ±0.1% Triton for intracellular staining. Staining with primary antibody (anti-GluR1 N-terminus; Millipore, monoclonal) was performed for 1 hour at room temperature at a concentration of 1∶50. Cells were washed 3 times for 10 minutes each, then stained with secondary antibody conjugated to Alexa Fluor 488 at a concentration of 1∶50. Cells were washed to remove unbound secondary antibody, then mounted with DAPI (Vectashield HardSet Mounting Medium with DAPI, Vector Labs). Slides were imaged using confocal microscope (Zeiss Axiovert 100, Zeiss Inc., NY).

### Intracellular Calcium Imaging

GBM BTICs were plated on 4 wells chambered coverslips (Lab-tek, Rochester, NY) previously coated with 10 µg/ml laminin. Prior to calcium imaging experiments, cells were grown in glutamine-depleted medium overnight. Cells were incubated with CG1-AM (Invitrogen-Life Technologies, Grand Island, NY) for 40 minutes (final concentration of 20 µM). Cells were then mounted on the stage of a Zeiss Axiovert 100 (Zeiss Inc., NY) washed with dye free HBSS and imaged to asses basal intracellular calcium levels. (S)-AMPA (Tocris), a selective prototypical agonist for AMPA receptors, and cyclothiazide (CYTZ, Tocris), a potent inhibitor of AMPA receptor desensitization, were dissolved in HBSS and added to the well to a final concentration of 100 µM for both agents. Calcium dependent changes in CG1 fluorescence lifetime were measured as described in Celli et al, 2010 [41]. Briefly, a Ti:Saph laser (Mira 900, Coherent, Santa Clara, CA) was used as the source of pulsed light. Fluorescence lifetime imaging (FLIM) measurements were performed using a TCSPC Becker and Hickl card (Becker and Hickl, Berlin, Germany). FLIM images were acquired at a rate varying from 3 frames per minute (fpm) to 1 fpm. FLIM data were analyzed using the phasor approach [42] and converted into calcium concentrations as described in Celli et al. and Sanchez et al [41], [43]. Only pixels with more than 100 photons were considered in the analysis. pf and pb (as defined in [41]) were obtained from FLIM measurements of cells exposed to 2.5 mM EGTA and 10 µM ionomycin or 5 mM Calcium and 10 µM ionomycin, respectively. A K_d_ = 76 nM [43] was used. Data are represented in false color calcium concentration images.

## Results

### Selective Culturing of GBM BTICs

To confirm that our BTIC cultures derived from the surgically resected GBM tissues were indeed concentrated in tumor stem cells, we performed immunoblotting assays using antibodies directed against two well known stem cell markers, nestin and Sox-2 [40]. BTIC and differentiated cultures from fresh GBM tissues were prepared as described in the Materials and Methods, and cell lysates were prepared from these cultures. Cell lysates were then subjected to immunoblotting assays and probed with nestin and Sox-2 specific antibodies. GAPDH level was used to normalize the protein amount.

**Figure 1:**
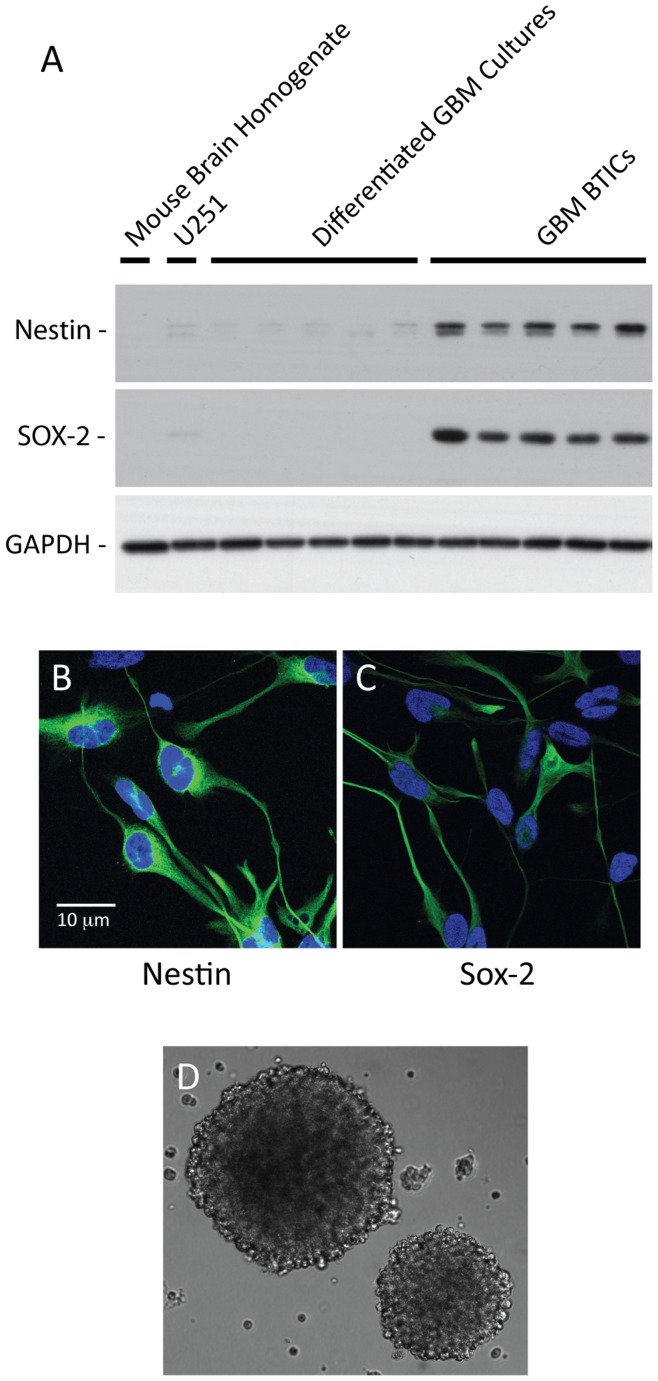
Confirmation of adherent GBM BTIC cultures. Adherent GBM BTICs were prepared and subjected to (A) immunoassays, (B and C) immunocytochemistry, and (D) plating on non-adherent conditions to confirm their stem cell properties. GBM BTICs strongly expressed neural stem cell markers, nestin and Sox-2, by both (A) immunoblotting and (B and C) immunocytochemistry. BTICs formed neurospheres when plated in non-adherent conditions (D).

Consistent with the previously reported study [40], BTICs derived from GBMs highly expressed nestin and Sox-2 compared to differentiated cultures derived from the same tumor tissues (Figure 1A). We used normal mouse brain homogenate and cell lysates from an established GBM cell line U251 as controls. Primary antibodies used could bind to both human and mouse proteins per manufactures’ specifications. While mouse brain homogenate did not express any detectable amounts of nestin or Sox-2, U251 cells expressed low levels of stem cell markers, which were similar to the differentiated GBM cultures. However, the stem cell markers in U251 and the differentiated GBM cultures were very low compared to BTICs. Expression of nestin and Sox-2 in GBM BTICs were also confirmed by immunocytochemistry using confocal microscopy (Figure 1B–C).

To further confirm that our BTIC cultures have stem cell-like properties, we grew these cells in non-adherent conditions in culture flasks not coated with laminin. Plating BTICs in non-adherent conditions promoted the growth of BTICs as neurospheres (Figure 1D). Finally, we injected suspended BTICs into athymic nude mice brains, which formed brained tumors, again confirming that our BTIC cultures do indeed have cancer stem cell properties (data not shown).

### Analysis of Glutamate Receptor Expression in Cultured GBM BTICs

To analyze the expression levels of AMPA-type glutamate receptors in BTICs compared to differentiated cultures derived from GBMs, we prepared cell lysates from BTICs and differentiated cultures derived from same GBM tumor tissues. We used mouse brain homogenate as a positive control and included cell lysates from established GBM cell line U251 as a comparison. This cell line has been previously shown to express AMPA receptor subunits [18], [39].

**Figure 2:**
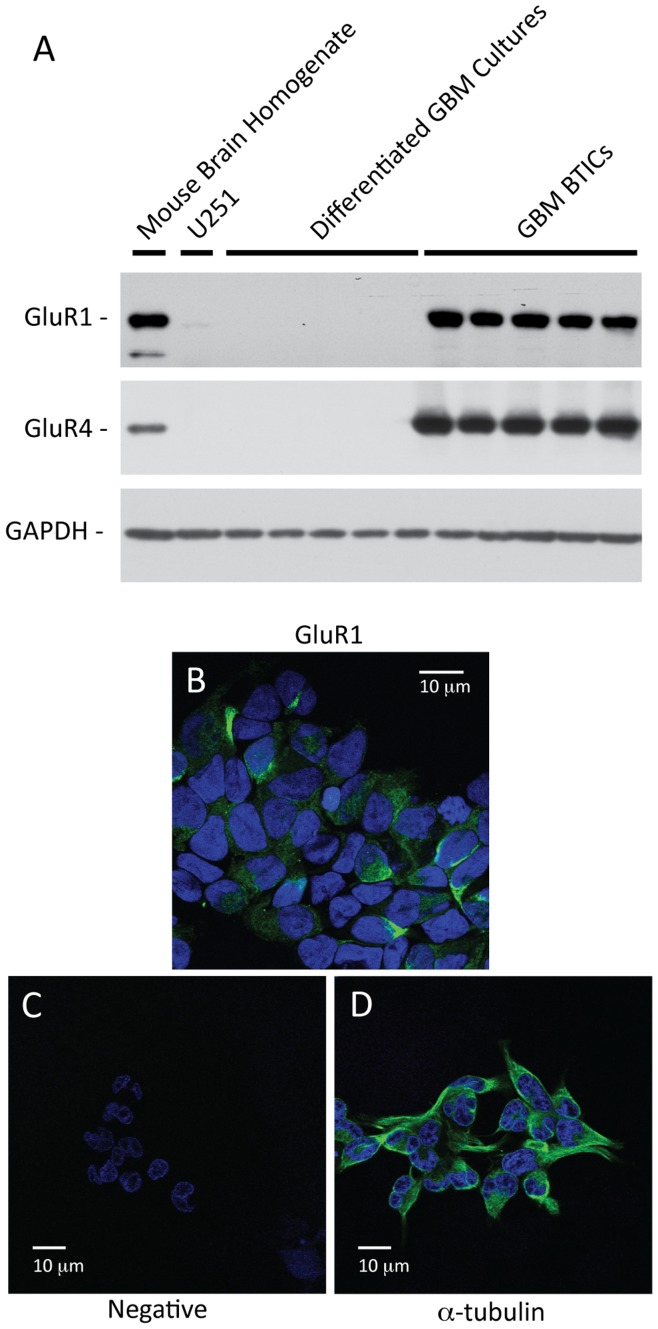
Expression of calcium-permeable AMPA receptor subunits in GBM BTICs. GBM BTICs strongly express calcium-permeable subunits of AMPA receptor (GluR1 and GluR4) compared to non-stem tumor cells (Primary GBM Cultures) and established GBM cell line U251 (A). Normal mouse brain homogenate (postive control) expressed both GluR1 and GluR4. To confirm that these calcium-permeable AMPA receptors are expressed on the surface membrane, GBM BTICs were stained with N-terminus GluR1 antibody under non-permeabilized condition (B). Compared to negative controls (C), GluR1 stained the surface membranes of BTICs. α-tubulin was stained after permeabilizing the membrane as a postive control (D).

Using AMPA receptor subunit specific antibodies, we performed immunoblotting assays and used GAPDH level to normalize the protein amount across different samples. Normal mouse brain homogenates strongly expressed GluR1 and GluR4, which are the calcium-permeable subunits of AMPA receptors (Figure 2A). While we detected low GluR1 expression in U251 cell lysates, GluR4 expression was absent. Moreover, both GluR1 and GluR4 expression levels in U251 cell line were quite low compared normal mouse brain homogenates. GluR2 subunit, which is present in calcium-impermeable AMPA receptor [44], was also not detectable in U251 lysates, even after concentrating for GluR2 subunit by immunoprecipitation, while expression was detectable in normal mouse brain homogenates (data not shown).

We compared GBM BTICs and differentiated cultures lysates derived from same tumor tissues to immunoblotting assays to compare the expression levels of calcium-permeable subunits, GluR1 and GluR4. As shown in Figure 2A, both GluR1 and GluR4 were strongly expressed in GBM BTICs. While GluR1 and GluR4 subunits were detectable in the differentiated cultures from the patient-matched tumor tissues, the blots needed to be over-exposed to detect their signal, suggesting very low expression levels (data not shown). Thus, GluR1 and GluR4 expression is clearly and consistently elevated in BTICs derived from GBM tumor tissues compared to the differentiated tumor cells. We also probed for the GluR2 subunit after immunoprecipitating with the GluR2 antibody, which was not detectable in either GBM BTICs or differentiated cultures (data not shown).

To confirm that AMPA receptors are expressed on the surface membrane, we performed immunocytochemistry using confocal microscopy after staining the GBM BTICs with the N-terminus (extracellular portion) GluR1 antibody under non-permeabilized condition. We saw avid staining for GluR1 subunits on the surface membrane of GBM BTICs (Figure 2B). We used μ-tubulin under permeabilized condition as a positive control (Figure 2D), while negative control consisted of no primary antibody (Figure 2C).

### Glutamate Receptors in GBM BTICs are Calcium-permeable

**Figure 3:**
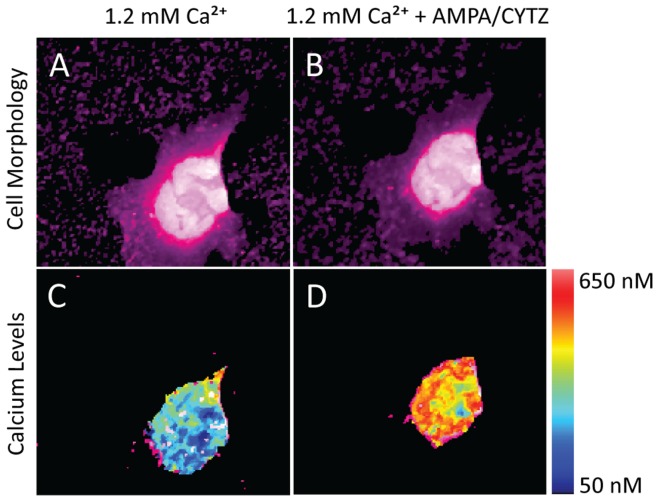
Intracellular calcium imaging in GBM BTICs in response to AMPA receptor stimulation. AMPA stimulation results in increased intracellular calcium levels in GBM BTICs. Cell morphology in 1.2 mM Ca^+2^ HBSS before and 10 minutes after AMPA and CYTZ stimulation showed no changes in cell morphology (A and B, respectively). Calcium levels 10 minutes after 100 µM AMPA and 100 µM CYTZ stimulation showed increase in intracellular calcium concentrations well exceeding 500 µM, suggesting that surface AMPA receptors on GBM BTICs are functional and conduct calcium currents (C and D, false color calcium images before and 10 minutes after calcium stimulation, respectively). The calcium concentrations color-coding is provided by the color scale on the right.

**Figure 4:**
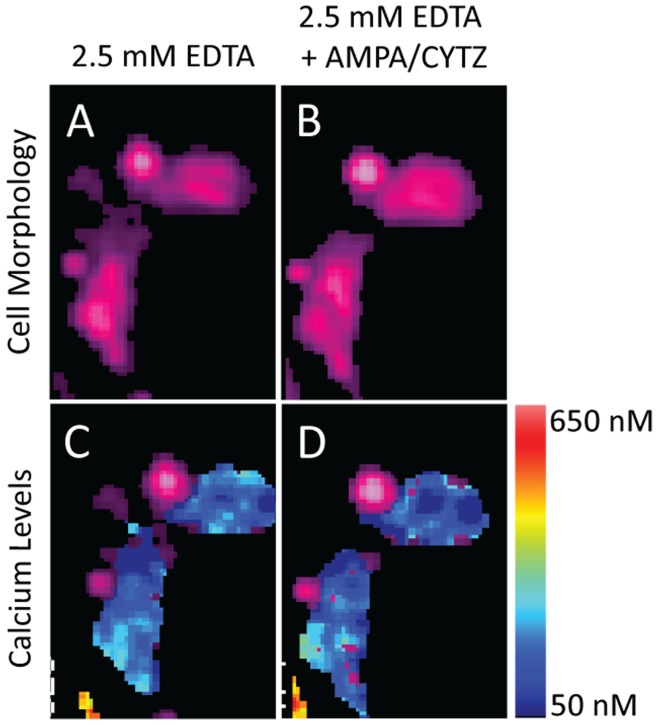
Intracellular calcium imaging in GBM BTICs in the absence of extracellular calcium. The source of increase in intracellular calcium levels is extracellular. Imaging in 2.5 mM EGTA and no extracellular calcium showed no change in intracellular calcium concentrations following 10 minutes of AMPA and CYTZ stimulation (C and D). Cell morphology in 2.5 mM EGTA with no extracellular calcium did not change throughout the experiment (A and B). The calcium concentrations color-coding is provided by the color scale on the right.

We performed intracellular calcium imaging using the CG1-AM calcium-sensitive dye to test whether surface AMPA receptors on GBM BTICs are functional. We stimulated the cells using bath application of 100 µM AMPA, a selective AMPA receptor agonist, and 100 µM CYTZ, an AMPA receptor desensitization inhibitor. Stimulation of GBM BTICs with AMPA and CYTZ resulted in prolonged increase in intracellular calcium concentration, sustained for more than 20 minutes, confirming that AMPA receptors on the surface of BTICs are functional and conduct calcium currents (Figure 3). To further confirm that the source of calcium was extracellular, we repeated the experiment in buffer with no calcium and 2.5 mM EGTA. Consistent with calcium-permeable AMPA receptors mediating the calcium signal, there were no changes in intracellular calcium concentration in response to AMPA and CYTZ under these conditions (Figure 4).

## Discussion

The first evidence for expression of glutamate receptors in glioma cells came from an electrophysiological study performed in *Xenopus* oocytes injected with mRNAs isolated from astrocytoma cell line R-111 [20]. Positive inward currents in response to glutamate and AMPA suggested that astrocytoma cells express AMPA-type glutamate receptors. Although no currents were elicited in oocytes injected with mRNAs from GBM cell lines in this study, a later study found that approximately two thirds of GBM cells express glutamate receptors, where currents in cultured GBM cells and acute slices made from tumor tissues in response to glutamate could be recorded [45]. Other studies have also confirmed the expression of AMPA receptors, but also NMDA, kainate, and metabotropic glutamate receptors in primary brain tumors [19], [21], [22]. Moreover, a study by de Groot and colleagues has shown that GBM cells express higher levels of GluR1 compared to anaplastic or low-grade astrocytomas, suggesting that level of GluR1 expression correlates with the grade of tumor [18]. Our study is the first to show the up-regulated expression of calcium-permeable AMPA receptors in GBM BTICs, when directly compared to non-stem tumor cells from patient-matched tumor tissues.

We used a previously described selective culturing technique to enrich adherent GBM tumor cells expressing stem cell markers [40]. Stem cell characteristics of GBM BTIC cultures were confirmed by the expression of stem cell markers, nestin and Sox-2, ability to form neurospheres, and ability to form differentiated tumors in xenotransplantation model, which are all hallmark features of BTICs [8], [40]. While this technique has limitations, the advantage of being able to stably culture an adherent BTICs is tremendous. A direct comparison of BTICs from non-BTICs from the same tumor tissue, for example, can give insights into key molecular differences that allow BTICs to act more aggressively, allowing tumor formation following xenotransplantation [7], [8]. Adherent GBM BTIC cultures also allows genetic and pharmacological manipulations to modulate and characterize key molecules involved in proliferation and invasion. These experiments can be followed by xenotransplantation experiments to see what role these molecules play in allowing BTICs to form tumors in animal models. One argument against the use of adherent GBM BTIC cultures is that protein expression may be falsely elevated due to EGF and FGF in the media. However, we have data suggesting that the expression levels of other key modulators of cellular proliferation (i.e. Akt and MAP kinase) are not different between GBM BTIC and differentiated GBM cultures (data not shown). Moreover, GluR2 subunit expression was undetectable in both cultures. Thus, we believe that up-regulated GluR1 and GluR4 expression in BTICs is a valid finding that warrants further studies to investigate the downstream effectors of these calcium-permeable AMPA receptors.

Although we did not test for expression of GluR3 subunit in BTICs, which has relatively low expression levels in glioma cells [21], [22], GluR2 subunit could not be detected in either BTIC or differentiated GBM cultures. By contrast, GluR2 signal could be detected on immunoblots with normal mouse brain homogenates. This is consistent with previous findings where only calcium-permeable AMPA receptor subunits were found in GBM cells [19], [36], [46], although low expression of GluR2 subunit was detectable in low-grade gliomas in one study [46]. In another study, GluR2 subunits were detected in malignant gliomas, but the post-transcriptional editing at the Q/R-site on the mRNA that makes GluR2 subunits calcium-impermeable was significantly under-edited, making these subunits calcium-permeable [47]. By contrast, nearly 100% of GluR2 subunits in the adult brain are edited at the Q/R-site, making them calcium-impermeable. Interestingly, under-editing of GluR2 was less severe in the low-grade astrocytomas [47]. Overall, these results together suggest that malignant gliomas tend to favor the expression of calcium-permeable AMPA receptors. Consistent with these findings, our results show up-regulated expression of calcium-permeable AMPA receptor subunits, GluR1 and GluR4, in GBM BTICs. The downstream effectors of these receptors and how their activity modulates GBM BTICs’ invasive potential is a subject of our future studies.

There is ample evidence currently to support the premise that glutamate receptors, and more specifically calcium-permeable AMPA receptors, do play a significant role in potentiating proliferation and invasion of GBM cells. Blocking glutamate receptor activity, for example, has been shown to block glioma proliferation [18], [19], [36], [48], [49], while direct pharmacological activation of glutamate receptors lead to increased invasion and proliferation [30], [35], [37]–[39]. Although the exact mechanism for this is unclear, some downstream effectors have been identified. The growth and motility of GBM cells following activation of calcium-permeable AMPA receptors has been shown to involve Akt activation via phosphorylation of Akt at serine 473 [36]. Moreover, AMPA receptor activation causes increase in EGF receptor expression in U-87 MG cell line, which is also linked to downstream phospho-Akt pathway [35]. Other key molecules downstream of phospho-Akt that mediate increased motility, proliferation, and invasion of GBM cells are yet to be determined.

The hypothesis that activation of calcium-permeable AMPA receptors in GBM BTICs allows them to proliferate and move into the surrounding normal brain tissues also needs to be confirmed in *in vivo* models. After migrating into normal brain tissues, BTICs can proliferate and differentiate, resulting in increase tissue glutamate concentration. This process may lead to further BTIC activation via calcium-permeable AMPA receptors, resulting in the highly aggressive phenotype of GBM.

While increased glutamate concentration in the tumor milieu may be one key factor in increasing GBM proliferation and invasion, how GBM cells survive this elevated excitotoxic levels of glutamate remains unclear. The U-87 MG GBM cell line, for examples, has been found to have at least 45 fold in resistance to glutamate excitotoxicity compared to normal glial cells [35]. A study by van Vuurden and colleagues found that GBM cells could tolerate high glutamate concentrations due to their down-regulated expression of AMPA receptor subunits compared to normal brain [50]. Our results showing that differentiated GBM cultures express very little GluR1 and GluR4 compared to the normal mouse brain support these findings. How GBM BTICs survive and thrive in such high glutamate concentration environment is also unclear. One possibility is that the kinetics of calcium currents in glioma cells may be very different than that of normal neurons and glial cells. For example, electrophysiological recordings of glioma cells with stimulation with glutamate or kainate elicited action potentials in some cells, while others have demonstrated depolarization with slower kinetics than one would expect from a fast acting excitatory ion channels [45]. In our study, we observed relatively slow increase in intracellular calcium in response to AMPA and CYTZ over time-scale of minutes, suggesting that kinetics of calcium currents are very different in GBM cells compared to normal neurons. Another possibility is that GBM cells may have significantly increased capacity to buffer intracellular calcium with different kinetics than normal neurons, allowing them to survive prolonged calcium influx. Overall, our results indicate that GBM BTICs do express high levels of calcium-permeable glutamate receptors and prolonged stimulation with AMPA and CYTZ, sometimes lasting up to 20 minute, can continue to elevate intracellular calcium concentrations. Factors involved in allowing GBM BTICs to survive the prolonged stimulation by increased glutamate concentration and calcium influxes could also lead to potential novel therapies.

Clinical trials are currently underway to determine whether glutamate receptor antagonist has any survival benefit for GBM patients. One particular drug called talampanel, an AMPA receptor antagonist, has undergone phase II clinical trial for newly diagnosed GBM patients. [51], [52] When compared to historical controls from the European Organisation for Research and Treatment of Cancer (EORTC), addition of talampanel to the standard radiation and temozolomide therapy prolonged the mean survival time from 14.6 months to 20.3 months and improved 24-month survival rate from 26.5% to 41.7% (p = 0.02). [51] Moreover, addition of talampanel as adjuvant therapy was well tolerated with minimal side-effects. However, these results should be taken with a caution, as talampanel as a single therapeutic agent for recurrent GBM patients had no significant effect on 6-month progression-free survival. [53] Currently, phase III clinical trials are underway for talampanel in addition to the standard radiation and temozolomide therapy for newly diagnosed GBM patients. Our results showing consistent and highly up-regulated expression of calcium-permeable glutamate receptors in GBM BTICs warrant further studies exploring the clinical benefits of glutamate receptor antagonists, and specifically calcium-permeable glutamate receptor antagonists, in GBM patients. Therapies specifically targeting GBM BTICs by blocking the calcium-permeable glutamate receptors may allow improved control of recurrences and disease progression following the surgical resection.

### Conclusion

Our results indicate that GBM BTICs overexpress calcium-permeable AMPA receptors, which conduct calcium influx in response to specific agonists. Presence of BTICs in GBM tissues with evidence supporting elevated glutamate concentrations in the tumor milieu warrant further studies exploring the role of increased intracellular calcium concentration mediated by calcium-permeable AMPA receptors in GBM BTICs.
